# Comparison of physical, mechanical and biological properties of a porcine urinary bladder acellular matrix collagen membrane with six other collagen membranes of animal origin

**DOI:** 10.1186/s12903-026-08397-1

**Published:** 2026-05-06

**Authors:** Fabio Camacho-Alonso, José Lacal-Luján, Ana María Mercado-Díaz, Marcos Rodríguez-Sánchez, Joaquín Sánchez, Alba Pérez Jardón, Jesús Pato Mourelo, Mario Pérez-Sayáns

**Affiliations:** 1 Department of Oral Surgery, University of Murcia, Murcia, Spain; 2 In private dental practice, Murcia, Spain; 3 Department of Histology and Pathological Anatomy, University of Murcia, Murcia, Spain; 4 Department of Oral Medicine, Oral Surgery and Implantology, Faculty of Medicine and Dentistry, Universidade de Santiago de Compostela, Santiago de Compostela, Spain; 5 Health Research Institute of Santiago de Compostela, ORALRES Group (IDIS), Santiago de Compostela, Spain; 6 In private dental practice, Sarria, Galicia Spain; 7 Materials Institute of Santiago de Compostela, (IMATUS) Universidade de Santiago de Compostela, Santiago de Compostela, A Coruña Spain; 8 Oral Surgery Teaching Unit, University Dental Clinic, Morales Meseguer Hospital, Marqués de los Vélez s/n, Murcia, 30008 Spain

**Keywords:** Acellular urinary bladder matrix, Collagen membranes, Guided bone regeneration, Osteoblast activity, Osteogenic potential

## Abstract

**Background:**

The porcine acellular urinary bladder matrix (AUBM) is a three-dimensional scaffold rich in collagens type I, III, IV, and VI that could be used for guided bone regeneration (GBR). The objective of this study was to compare the physical, mechanical and biological properties of a AUBM with six other collagen membranes of porcine, equine and bovine origin.

**Methods:**

Seventy membranes were included (*n* = 10 per group). The properties of AUBM membrane were compared with the other animal collagen membranes available: porcine pericardium (PP), equine pericardium (EP), bovine pericaridium (BP), porcine peritoneum (PPE), equine Achilles tendon (EAT), bovine Achilles tendon (BAT). We analysed physical properties (surface morphology, hydrophilic property, degradation ratio and thermal stability), mechanical properties (tensile strength and dry and wet elongation), and biological properties (cell viability, quantification of type I collagen and osteopontin, Alkaline Phosphatase (ALP) activity and calcium deposition).

**Results:**

AUBM membranes showed a surface morphology (in smooth and rough surfaces) very similar to those of PP, EP, EAT, and BAT. The most hydrophilic membrane was PPE. The AUBM membranes showed a low degradation ratio and thermal stability similar to the other membranes. AUBM membranes had a tensile strength (dry and wet) and elongation (dry) similar to the other membranes. Their elongation was much higher than the rest when wet. AUBM membrane showed good biological properties.

**Conclusions:**

AUBM membrane showed physical and mechanical properties similar to the other six membranes. However, upon wetting they were the membranes with the highest elongation capacity and showed similar properties to PPE membranes in promoting osteogenesis. AUBM could be an ideal collagen source for the manufacture of membranes for GBR, but further clinical studies are needed.

## Background

Dental implants are widely used for tooth rehabilitation [1, 2], but the successful placement of implants can be hindered by inadequate alveolar bone, necessitating bone regeneration techniques [3–9]. Guided Bone Regeneration (GBR), a method using physical barriers such as collagen membranes, promotes bone healing by preventing unwanted tissue infiltration while allowing bone-forming cells to regenerate the defect [10–12].

Membranes for GBR are typically classified as resorbable or non-resorbable. While resorbable membranes offer fewer postoperative complications, their regeneration potential is limited by their degradation rates [13]. In contrast, non-resorbable membranes provide better structural support but come with higher complication rates and require a second surgical procedure for removal [14–19]. Resorbable membranes, (e.g., chitosan or collagen), are widely used in GBR, although their biological response depends on the polymer used [20–28]. Collagen membranes are particularly useful due to their biocompatibility, flexibility, and ability to promote cell adhesion [29–32]. These membranes are derived from various animal tissues, including porcine, equine, and bovine sources [33–38].

Despite their widespread use, current collagen membranes often have limitations, such as insufficient elongation capacity when wet, variable degradation rates, and suboptimal osteogenic potential [32, 39–43]. Acellular Urinary Bladder Matrix (AUBM), a novel collagen source rich in collagen types I, III, IV, VI, elastin, and other bioactive molecules, has shown promising potential for soft tissue reinforcement and wound healing [44–50]. However, its properties for GBR have not been thoroughly evaluated.

This study aims to compare the physical, mechanical, and biological properties of AUBM with those of other commonly used collagen membranes to assess its suitability for GBR applications.

## Methods

### In vitro study design

This study was conducted in accordance with the guidelines outlined in the Checklist for Reporting In-vitro Studies (CRIS) [51]. It received approval from the Biosafety Committee on Experimentation of the University of Murcia (594/2023) and was carried out between May 2023 and February 2024 at the University Dental Clinic and the Research Support Service of the University of Murcia, and in the Biomaterials, Biomechanics and Tissue Engineering Group (BBT) of Polytechnic University of Cataluña.

### Collagen membrane materials

To compare the physical, mechanical and biological characteristics of a AUBM collagen membrane with other animal collagen membranes on the market, we selected a total of six types of heart and non-heart collagen membranes of porcine, equine and bovine origin. Heart: porcine pericardium (PP), equine pericardium (EP), bovine pericardium (BP). Non-heart: porcine peritoneum (PPE), equine Achilles tendon (EAT), bovine Achilles tendon (BAT), and the AUBM collagen membrane (AUMB) (Table 1). A total of 10 membranes were used for this in vitro study, 5 to analyse the physical and mechanical properties and 5 to analyse the biological properties.

**Table 1:** Collagen membranes used for the study

Collagen Source	Brand	Manufacturer	LOT	Constituent	Abbreviations
Heart					
Porcine pericardium	BoneProtect^®^	Dentegris Deutschland GmbH, Duisburg, Germany	22GA51180	Heterologous collagen	PP
Equine pericardium	HEART^®^	Bioteck S.p.A., Arcugnano, Italy	234004	Heterologous collagen	EP
Bovine pericardium	Lyoplant^®^	B.Braun Biotech International, Melsungen, Germany	223373	Heterologous collagen	BP
Non-Heart					
Porcine peritoneum	Bio-Gide^®^	Geistlich Pharma AG, Wolhusen, Switzerland	82300440	Heterologous collagen	PPE
Equine Achilles tendon	Biocollagen^®^	Bioteck S.p.A., Arcugnano, Italy	223871	Heterologous collagen	EAT
Bovine Achilles tendon	COLLA^®^	MedPark Co, Ltd., Busan, Republic of Korea	BS220425A	Heterologous collagen	BAT
Porcine urinary bladder	----------	----------	----------	Heterologous collagen	AUBM

### Preparation of AUBM collagen membrane

**Fig. 1:**
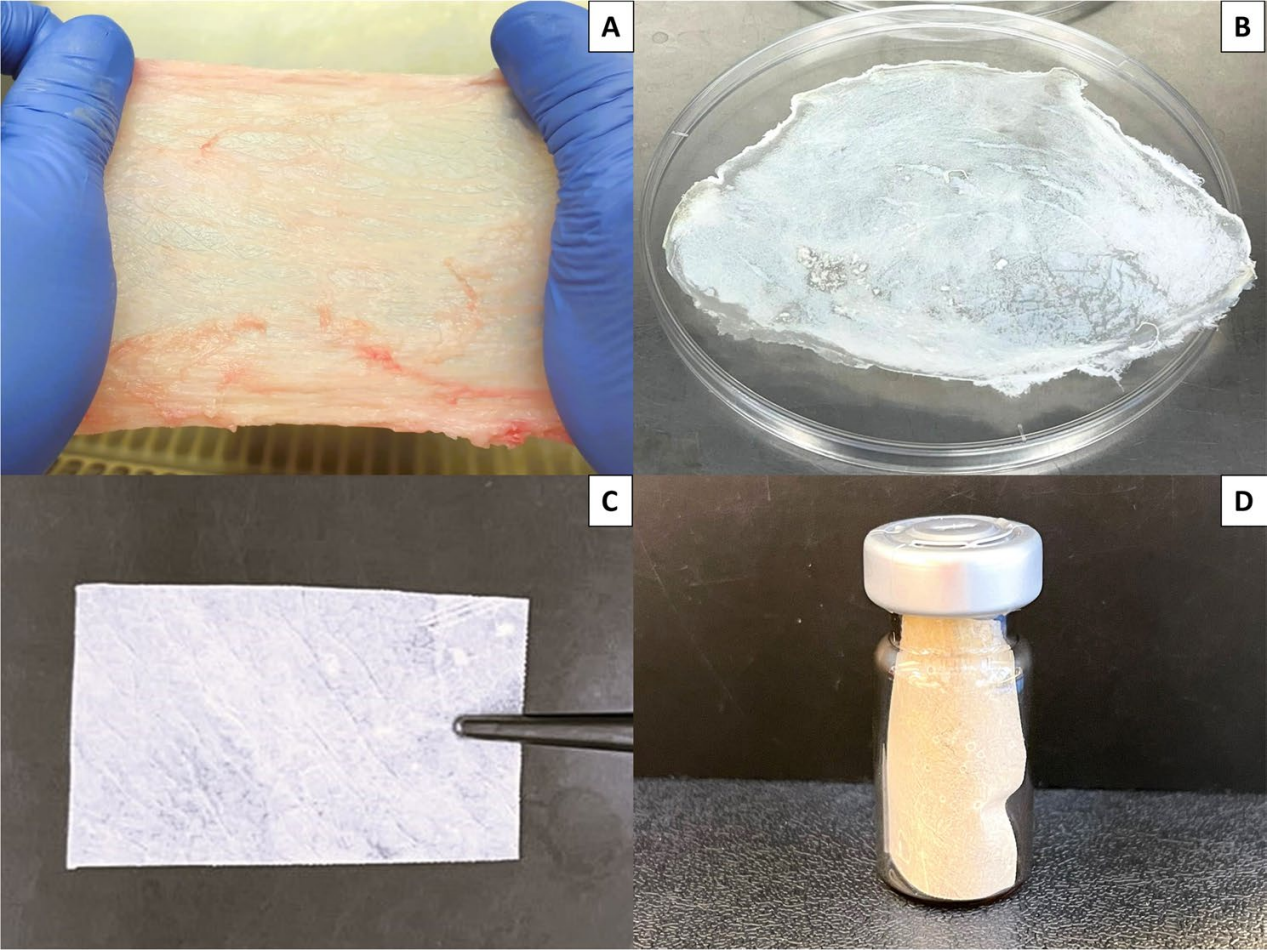
Preparation of the collagen membrane of AUBM. **A**: AUBM obtained from the lamina propria of porcine bladder, following the elimination of serous and muscular tunics. **B**: Lamina propria after decellularisation process. **C**: AUBM collagen membrane of 20 x 30 mm. **D**: New collagen membrane of AUBM after sterilisation

**Fig. 2:**
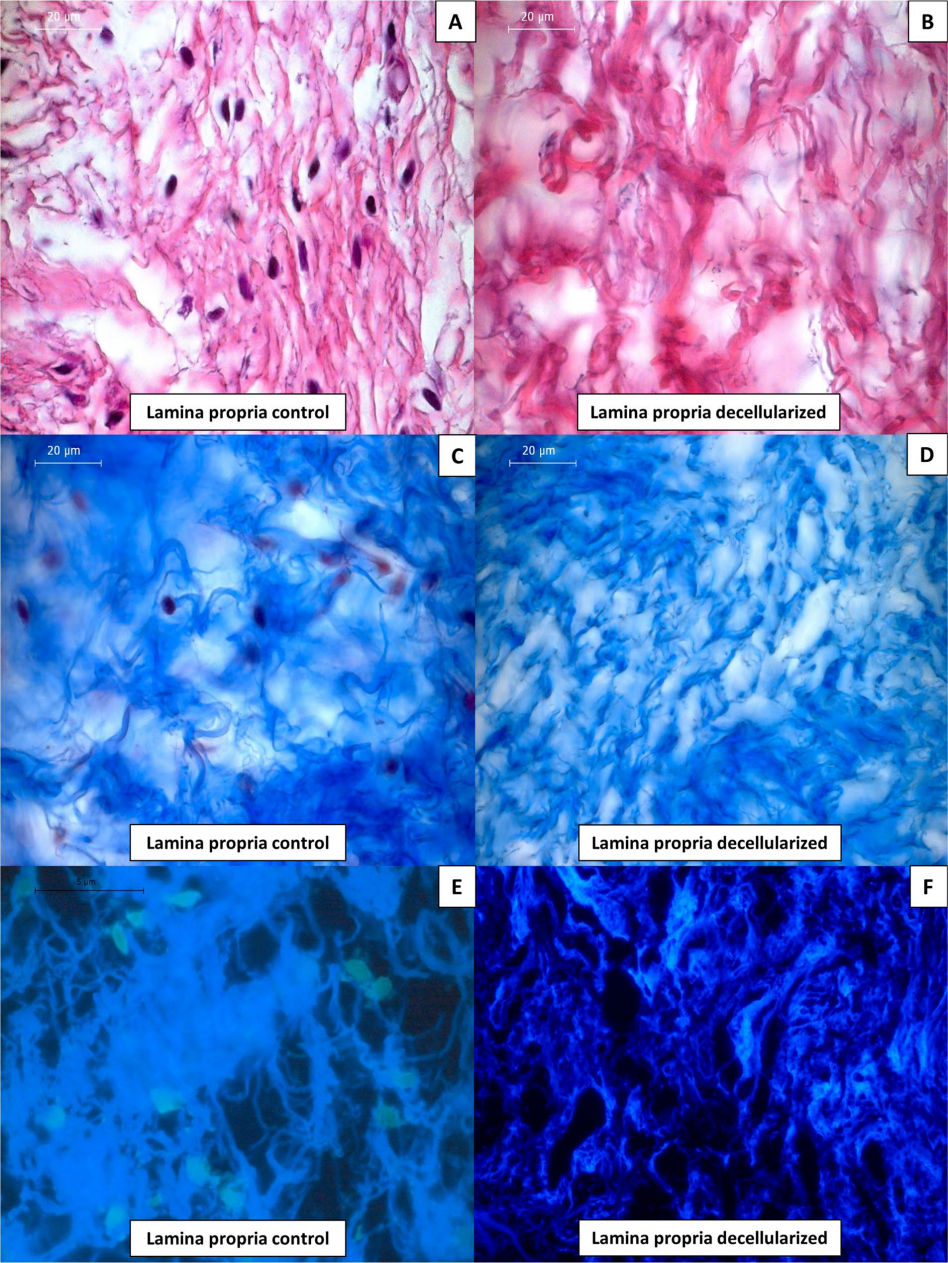
Comparison of cellular element absence between the control lamina propria and decellularised lamina propria. **A** & **B**: Haematoxylin and eosin staining images at 500x. **C** & **D**: Masson’s trichrome staining images at 500x. **E** & **F**: DAPI staining images at 500x

AUBM collagen membranes were fabricated from the porcine urinary bladder of four male pigs, with weights between 147 and 155 kg (mean weight 150 kg), as described in the literature [52, 53]. The urinary bladders came from pigs slaughtered in the Orihuela, Murcia, Spain abattoir (Registry number ES030990000631). The bladder was cleaned and trimmed to obtain the lamina propria (Fig. 1A), which was placed in 0.05% trypsin (Sigma-Aldrich Chemistry, S.A., Madrid, Spain) for 1 h at 37 °C. Then, it was placed in a solution of Dulbecco’s modified eagle medium containing 10% phosphate buffered saline and 1% antibiotic/antimycotic, and stored overnight at 4 °C. On the subsequent day, the preparation was washed in a solution of 1% Triton X (Sigma-Aldrich Chemistry, S.A., Madrid, Spain) and 0.1% ammonium hydroxide (Fisher Scientific, S.L., Madrid Spain) in deionized water for 4 days at 4 °C. It was then rinsed in deionized water for 3 days at 4 °C. To ensure the absence of cellular elements and the proper preservation of structural components, a portion of lamina propria was removed from the decellularization process and used as a control sample. Both were histologically evaluated using haematoxylin and eosin (Fig. 2A & B), Masson’s trichome staining (Fig. 2C & D), and 4 × 6-diamino-2-phenylindole dihydrochloride, D952 (DAPI) (Sigma-Aldrich Chemistry, S.A., Madrid, Spain). The DAPI-stained samples with were observed under a Leica^®^ DRMB fluorescence microscope (Leica Microsystems, Wetzlar, Germany) equipped with DAPI band filter (wavelength excitation filter set at BP 340–380, dichroic filter RKP 400 and emission filter LP425) (Fig. 2E & F). The decellularized lamina propria was then desiccated using a Rotovapor (Büchi, Dlawil, Switzerland) (Fig. 1B). Finally, they were cut into 20 × 30 mm collagen membranes (Fig. 1C) and sterilised at 25 kGy of gamma radiation (Fig. 1D).

### Physical properties 1: surface morphology with field emission scanning electron microscopy (FESEM)

A total of 25 samples from each group (5 portions of the 5 membranes per group) were used. The samples were subjected to platinum sputtering with a thin layer (5.0 nm) using a Leica ACE600 (Leica Microsystems, Heidelberg GmbH, Mannheim, Germany) and the structure of the smooth and rough surfaces was examined by field emission scanning electron microscopy (FESEM) (ApreoS Lovac IML, Thermo Fisher Scientific, Waltham, MA, USA), with a selected voltage of 5 kV. Samples of each surface were photographed at 200X and 500X. Following the different morphologies of collagen surfaces described by Shi et al., [34], the smooth and rough surfaces were classified as: collagen bundles were regular and closely arranged, slightly irregular and loosely arranged, or interwoven together other to form a three-dimensional (3D) grid with consistent pores.

### Physical properties 2: hydrophilic property

A total of 25 samples from each group (5 portions of the 5 membranes per group) were used. The hydrophilicity of the smooth and rough surfaces of the membranes was assessed by measuring the contact angles. To sum up, 3 µL of distilled H_2_O was placed on the collagen membrane surface, and the contact angle was measured after 4 s using an optical contact angle measuring instrument DataPhysics model OCA 15 (DataPhysics, Filderstadt, Germany) equipped with image analysis software SCA20 (Dataphysics, Filderstadt, Germany) at room temperature. This device makes it possible to quantify the interaction between the adhesive forces between the liquid and the solid and the cohesive forces of the liquid. When the adhesive forces to the solid surface exceeded the cohesive forces, the contact angle is below 90º, indicating a higher hydrophilic capacity of the collagen membranes.

### Physical properties 3: degradation ratio assay

The seven types of collagen membranes were cut into circles with a diameter of 6 mm (Fig. 5A) and transferred to a 15-mL centrifuge tube. Artificial saliva (10 mL) (Sigma-Aldrich Chemistry, S.A., Madrid, Spain) was subsequently introduced with a sterilised filter (10 specimens in each group). The samples were then incubated at 37 °C and 130 rpm. The residual specimens were extracted on days 7, 14, and 21, then freeze-dried and weighed [54] (Fig. 5B). The degradation rate was expressed as the percentage of weight loss, calculated by the following formula: Weight loss (%) = (W_0_ - W_d_) x 100/W_0_, where W_0_ is the weight before degradation and W_d_ is the dry wight after degradation.

### Physical properties 4: thermal stability analysis

A total of 3 samples from each group were used. Thermogravimetry was performed on the thermal analyser SDT 650 (TA Instruments, New Castle, USA) calibrated with indium, zinc, gold and sapphire. The analyses were performed under oxygen atmosphere, with a flow rate of 100 mL/min from room temperature to 800 °C, with a heating rate of 10 °C/min. Data were analysed using the software TRIOS (TA Instruments, New Castle, EEUU) and expressed as weight (%) for the TG curve (weight loss as a function of temperature).

### Mechanical properties 1 and 2: tensile strength and elongation

A total of 10 samples from each group (2 portions of the 5 membranes per group) were used, 5 for dry testing and 5 for wet testing. Membranes in the wet test were immersed in artificial saliva (Sigma-Aldrich Chemistry, S.A., Madrid, Spain) for 2 min before testing. Each of the samples were 20 mm x 5 mm and mounted on the gripping unit of the tester at room temperature and the tensile strength was calculated using a universal mechanical testing machine Bionix 358 (Bionix, Massachusetts, USA) with a 500 N cell load. Tensile force was exerted at a crosshead speed of 5 mm/min until the sample fractured. The calculations for tensile strength and elongation at break were performed using these formulas: Tensile strength (MPa) = Maximum load at break (N)/Cross-sectional area (m^2^) [55] and Elongation (%) = Extension of length at break x 100/Initial length [56].

### Biological properties 1: cell viability (cell proliferation assay)

A total of 5 samples from each group (1 portion of the 5 membranes per group) were used. The seven types of membranes were cut into circles (8 mm diameter) and transferred to a 96-well culture dishes. Human osteoblasts hFOB 1.19 cells CRL-113TM (ATTC, VA, USA) at a density of 1 × 10^3^ cells per well were seeded on the membranes. Cell proliferation was assessed at 24, 48, and 72 h of incubation by the (3-[4,5-dimethylthiazol-2-yl]-2,5 diphenyl tetrazolium bromide) (MTT) assay. First, 20 µL of MTT (Sigma-Aldrich Química S.A., Madrid, Spain) at a concentration of 5 mg/mL was added to 180 µL of medium to give a final concentration of 1 mg/mL and incubated for 4 h, then the medium was removed and 100 µL dimethyl sulfoxide (DMSO) (Sigma-Aldrich Química S.A., Madrid, Spain) was added to each well. Results were analysed by measuring absorbance at a wavelength of 570 nm using a FLUOstar Omega plate reader (BGM LabTech, Ortenberg, Germany). All experiments were performed in blind, biological triplicate.

### Biological properties 2 and 3: quantification of type I collagen and osteopontin

After 3, 5 and 7 days, type I collagen and osteopontin secreted by the cells cultured on the various surfaces were measured by enzyme-linked immunosorbent assay (ELISA), for type I collagen we used Human Collagen Type I ELISA Kit AB285250 (Abcam Inc., Cambridge, UK), and for osteopontin we used Human Osteopontin ELISA Kit (Sigma-Aldrich Química S.A., Madrid, Spain). The supernatant was aspirated and centrifuged at 5000 g for 15 min at 4 °C, and aliquots from each sample were analysed using ELISA to determine the levels of type I collagen and osteopontin, following the manufacturer’s conditions. Results were analysed by measuring absorbance at a wavelength of 570 nm using a FLUOstar Omega plate reader (BGM LabTech, Ortenberg, Germany). All experiments were performed in blind, biological triplicate.

### Biological properties 4: ALP activity

Human osteoblasts hFOB 1.19 cells CRL-113TM (ATTC, VA, USA) were seeded at a density of 5 × 10^4^ on top of the matrix for 7 days. The colorimetric ALP assay kit AB83369 (Abcam Inc., Cambridge, UK) was employed to evaluate ALP levels, according to the manufacturer’s conditions. The results were analysed by measuring absorbance at a wavelength of 570 nm using a FLUOstar Omega plate reader (BGM LabTech, Ortenberg, Germany). All experiments were performed in blind, biological triplicate.

### Biological properties 5: alizarin red staining (calcium deposition)

Human osteoblasts hFOB 1.19 cells CRL-113TM (ATTC, VA, USA) were seeded at a density of 5 × 10^4^ on top of the matrix for 14 days. The Osteogenesis Quantification Kit EMC815 (Sigma-Aldrich Química S.A., Madrid, Spain) was used to assess calcium deposition levels, according to the manufacturer’s conditions. Results were analysed by measuring absorbance at a wavelength of 570 nm using a FLUOstar Omega plate reader (BGM LabTech, Ortenberg, Germany). All experiments were performed in blind, biological triplicate.

### Statistical analysis

Data were analysed using SPSS 20.0 statistical programme (SPSS^®^ Inc, Chicago, IL, USA). A descriptive study was performed for each variable. Associations between qualitative variables were studied using Pearson’s chi-squared test. ANOVA and Tukey tests were applied to quantitative variables, determining in each case whether the variances were homogeneous. Statistical significance was considered for *p* < 0.05.

## Results

### Physical properties 1: surface morphology with field emission scanning electron microscopy (FESEM)

**Fig. 3:**
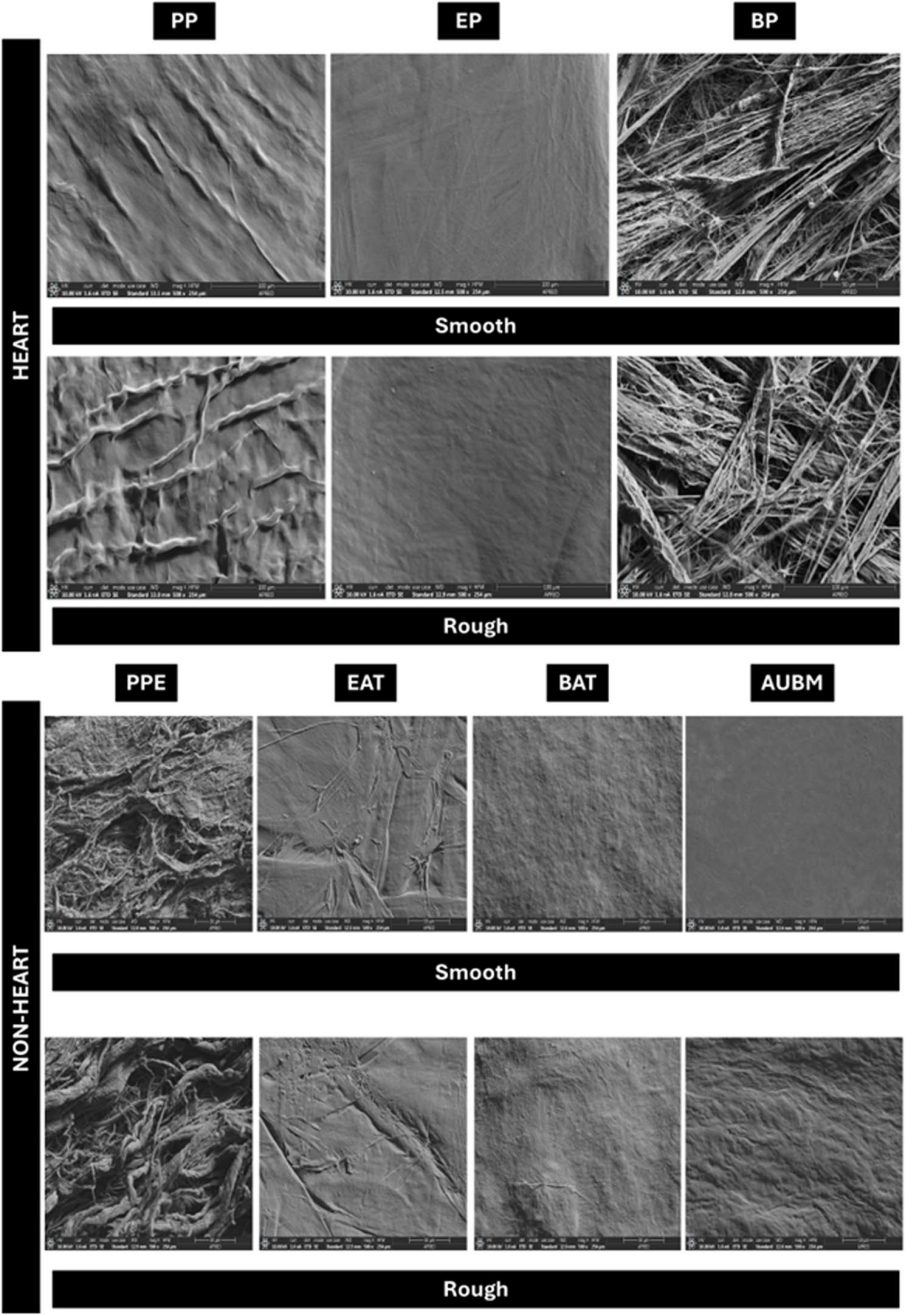
Surface morphology with FESEM of heart and non-heart collagen membranes at 500x

The AUBM collagen membrane showed on both surfaces (smooth and rough) regular and closely arranged collagen bundles, as did the PP, EP, EAT and BAT membranes. The PPE collagen membranes showed on both surfaces slightly irregular and loosely arranged collagen bundles, and BP membranes showed collagen bundles interwoven on both surfaces, forming a 3D grid with uniform pores (Fig. 3).

### Physical properties 2: hydrophilic property

The contact angle (the smaller the angle the higher the hydrophilic capacity) of the collagen membranes on both surfaces (smooth and rough) in order from smallest to largest was PPE < AUBM < EP < BP < BAT < PP < EAT. The PPE collagen membranes were the most hydrophilic with statistically significant differences (*p* ≤ 0.050) with respect to the other membranes studied. In fact, the contact angle of the drop of distilled H_2_O was not able to be measured after 4 s on any of the surfaces, because it was absorbed in less than 4 milliseconds.

**Fig. 4:**
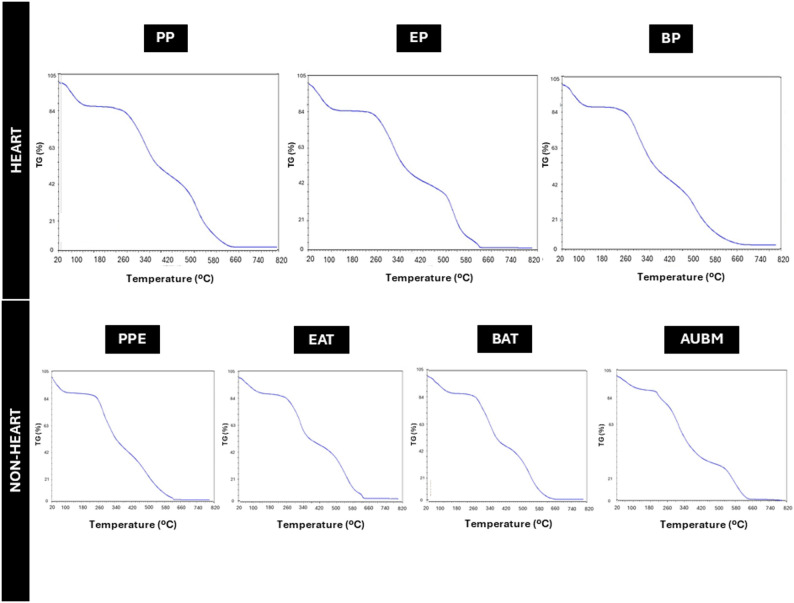
Water contact angle of collagen membranes

The collagen membrane of AUBM showed in both surfaces (smooth and rough) to be more hydrophilic than PP, EP, BP, EAT and BAT, with statistically significant differences with PP (*p* < 0.001 in both surfaces), EAT (*p* < 0.001 in both surfaces) and BAT (*p* = 0.007 in smooth surface and *p* = 0.005 in rough surface) (Table 2 & Fig. 4).

**Table 2:** Comparison of the hydrophilic property (Tukey test)

Collagen membranes	Contact angles (smooth surface) mean ± SD	Contact angles (rough surface) mean ± SD
PP	99.55 ± 17.91^b, c,d, g^	99.35 ± 19.91b, c,d, g
EP	66.01 ± 14.73^a, d,e^	59.69 ± 12.25^a, d,e^
BP	66.98 ± 23.52^a, d,e^	62.19 ± 23.02^a, d,e^
PPE	0 ± 0^a, b,c, e,f, g^	0 ± 0^a, b,c, e,f, g^
EAT	103.96 ± 3.81^b,c,d,g^	99.25 ± 9.62^b,c,d,g^
BAT	84.81 ± 5.85^d, g^	79.02 ± 9.31^d, g^
AUBM	43.99 ± 10.56^a, d,e, f^	42.64 ± 9.41^a, d,e, f^

^a^ Significant differences compared with PP

^b^ Significant differences compared with EP

^c^ Significant differences compared with BP

^d^ Significant differences compared with PPE

^e^ Significant differences compared with EAT

^f^ Significant differences compared with BAT

^g^ Significant differences compared with AUBM

### Physical properties 3: degradation ratio assay

The degradation weight loss (%) of the membranes at 7, 14 and 21 days ranked from lowest to highest was AUBM < PPE < EP < BP < BAT < PP < EAT. The collagen membranes of EAT showed the highest degradation ratio at 7, 14 and 21 days, with statistically significant differences at 7 days with AUBM (*p* = 0.024), at 14 days with EP, BP, PPE, BAT and AUBM (*p* < 0.001), and at 21 days with EP, BP, PPE and AUBM (*p* < 0.001).

**Fig. 5:**
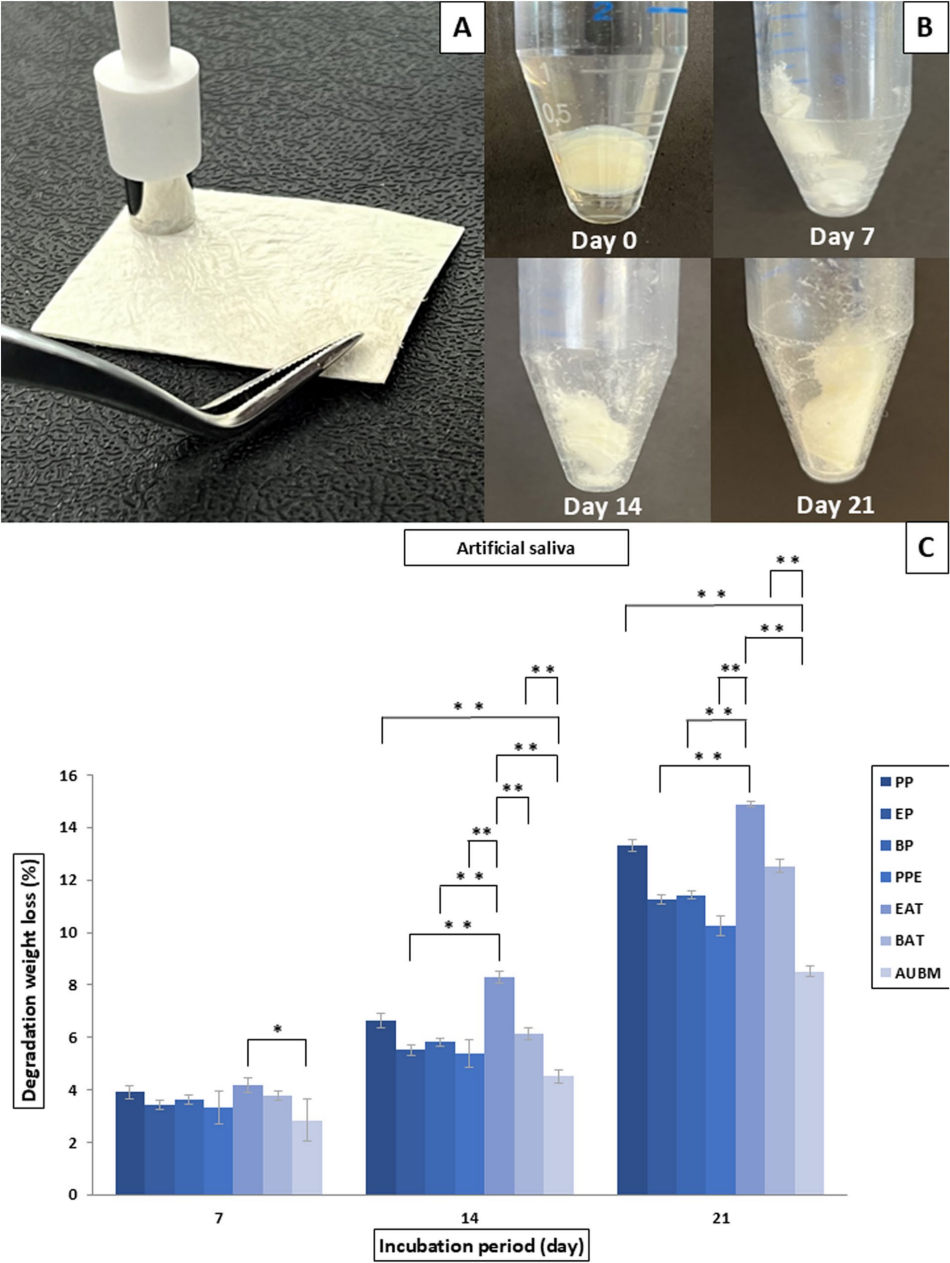
Degradation ratio assay. **A**: Collagen membranes cut into circles using a 6 mm diameter circular scalpel. **B**: Photographs of collagen membrane of AUBM before 7, 14 and 21 days after degradation with artificial saliva. **C**: Degradation weight loss (%) of membranes incubated with artificial saliva (* *p*<0.05; ** *p*<0.001)

The collagen membrane of AUBM showed the lowest degradation ratio at 7, 14 and 21 days, with statistically significant differences at 7 days with those of EAT (*p* = 0.024), and at 14 and 21 days with those of PP, EAT and BAT (*p* < 0.001) (Fig. 5C).

### Physical properties 4: thermal stability analysis

**Fig. 6:**
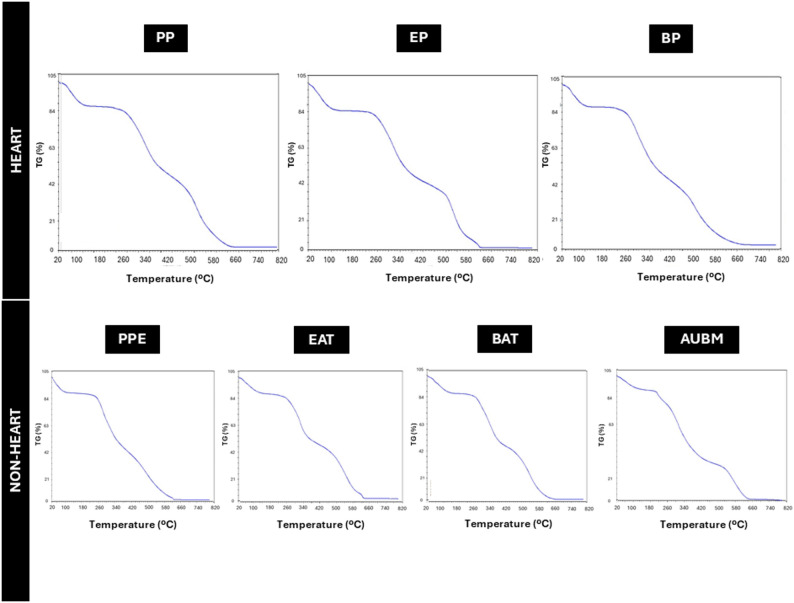
TG curves of collagen membranes

The TG curves of weight loss (%) as a function of temperature were very similar for the seven membranes studied, with three temperature peaks showing the highest percentage weight loss for all membranes at 80º C, 300º C and 550º C (Fig. 6). When comparing weight loss (%) at these three temperature peaks, they were very similar for all membranes, with no statistically significant differences (Table 3). Finally, all membranes were totally destroyed at 660 °C and above.

**Table 3:** Thermal stability analysis (Tukey test)

Collagen membranes	Weight loss (%) at 80º C mean ± SD	Weight loss (%) at 300º C mean ± SD	Weight loss (%) at 550º C mean ± SD
PP	14.12 ± 2.11	40.84 ± 2.14	72.91 ± 3.79
EP	16.23 ± 1.36	43.41 ± 3.16	69.35 ± 2.68
BP	13.42 ± 2.03	43.92 ± 4.68	68.91 ± 3.46
PPE	12.81 ± 1.06	45.86 ± 2.79	71.68 ± 5.23
EAT	13.05 ± 2.08	40.51 ± 4.16	73.26 ± 4.67
BAT	13.15 ± 1.09	42.34 ± 5.67	72.57 ± 5.26
AUBM	10.66 ± 2.07	59.44 ± 2.68	71.56 ± 3.68

### Mechanical properties 1 and 2: tensile strength and elongation

**Fig. 7:**
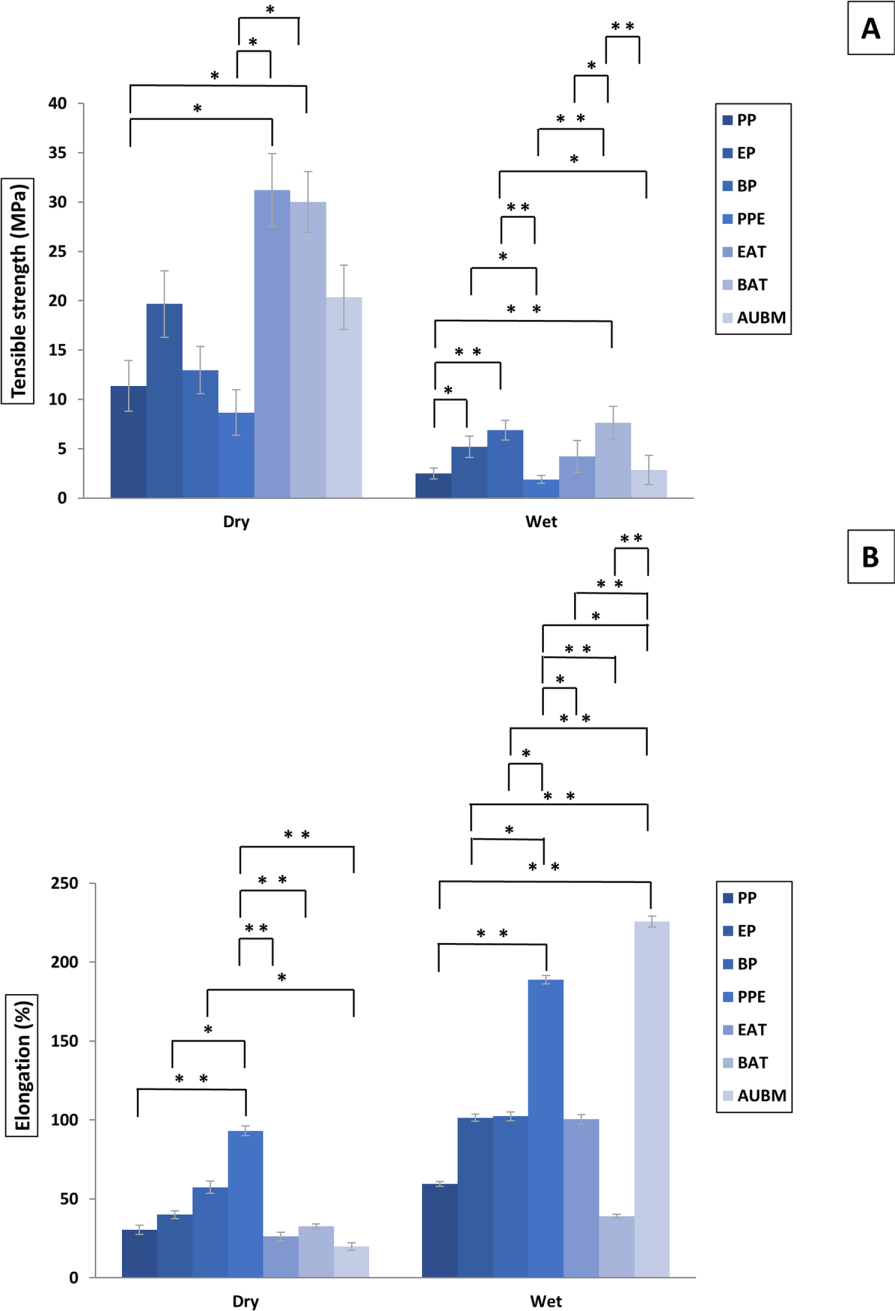
**A**: Tensile strength (* *p*<0.05; ** *p*<0.001). **B**: Elongation (* *p*<0.05; ** *p*<0.001)

The tensile strength (MPa) of the dry collagen membranes, listed from highest to lowest, was EAT > BAT > AUBM > EP > BP > PP > PPE. The EAT membranes showed the highest dry tensile strength, with statistically significant differences with PP (*p* = 0.032) and PPE (*p* = 0.022). The collagen membrane of AUBM was the third highest in dry tensile strength, with no statistically significant differences with any of the other membranes (Fig. 7A).

The tensible strength of the wet collagen membranes was lower than dry in all the membranes, with the descending order being BAT > BP > EP > EAT > AUBM > PP > PPE. The BAT membranes showed the highest wet tensile strength, with statistically significant differences with PP (*p* < 0.001), PPE (*p* < 0.001), EAT (*p* = 0.007), and AUBM (*p* < 0.001). The collagen membrane of AUBM was the fifth highest in wet tensile strength (Fig. 7A).

The elongation (%) of the dry collagen membranes, arranged in descending order, was PPE > BP > EP > BAT > PP > EAT > AUBM. The PPE membranes showed the highest dry elongation capacity, showing statistically significant differences with PP (*p* < 0.001), EP (*p* = 0.001), EAT (*p* < 0.001), BAT (*p* < 0.001), and AUBM (*p* < 0.001) (Fig. 7B).

**Fig. 8:**
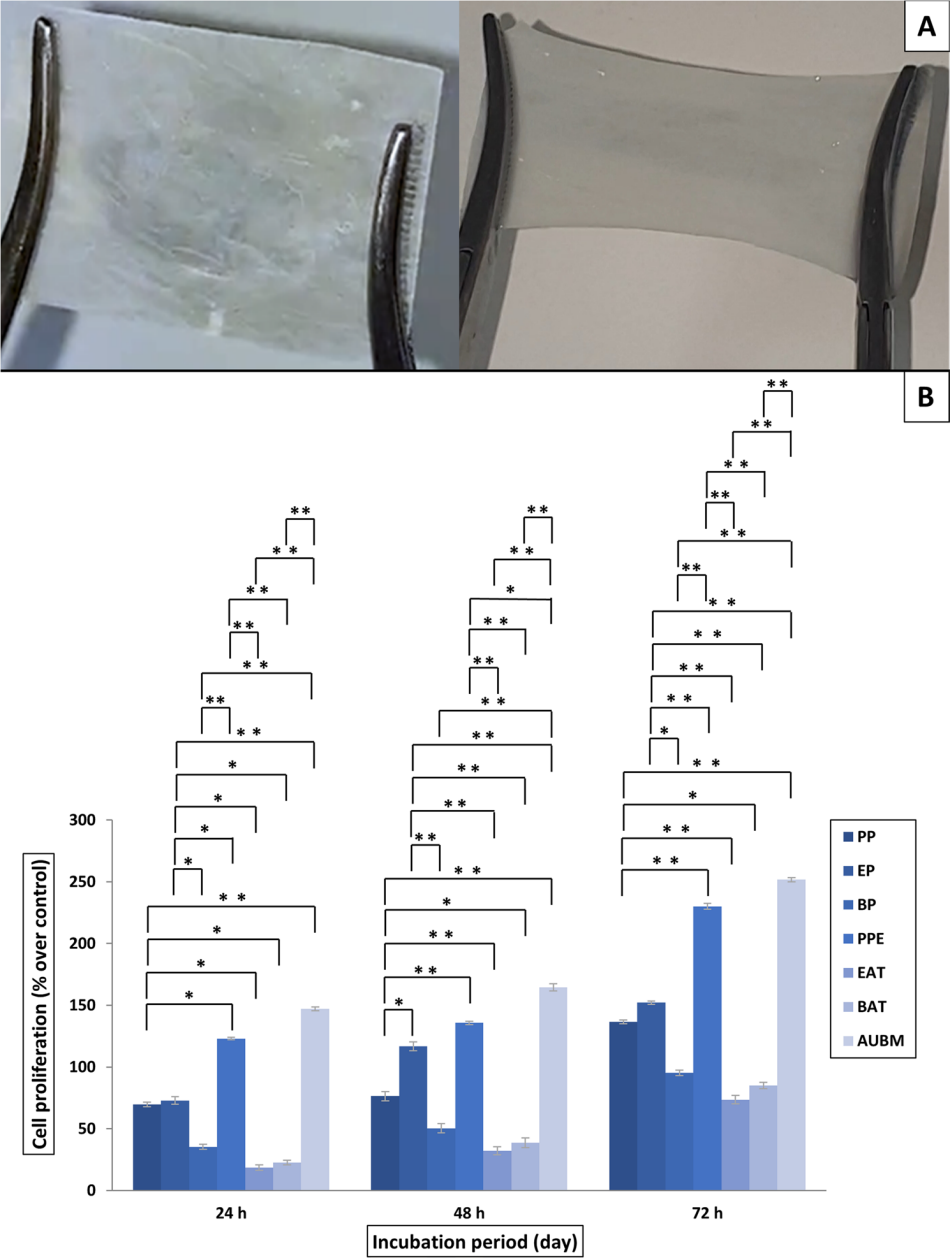
**A**: Increase in tensile strength of the new collagen membrane after soaking in artificial saliva for 2 min. **B**: Cell proliferation at 24, 48 and 72 h of incubation

The elongation of the wet collagen membranes higher than in dry in all membranes, the order from highest to lowest was AUBM > PPE > BP > EP > EAT > PP > BAT. The collagen membrane of AUBM showed the highest wet elongation capacity, with statistically significant differences with all the rest: PP (*p* < 0.001), EP (*p* < 0.001), BP (*p* < 0.001), PPE (*p* = 0.019), EAT (*p* < 0.001), and BAT (*p* < 0.001) (Fig. 7B & 8A).

### Biological properties 1: cell viability (cell proliferation assay)

Cell proliferation at 24, 48 and 72 h of incubation (expressed as a percentage relative to the control) of the collagen membranes ranked as follows: AUBM > PPE > EP > PP > BP > BAT > EAT; observing statistically significant differences at 24 h of incubation of the collagen membrane of AUBM with PP (*p* < 0.001), EP (*p* < 0.001), BP (*p* < 0.001), EAT (*p* < 0.001), y BAT (*p* < 0.001); at 48 h with PP (*p* < 0.001), EP (*p* < 0.001), BP (*p* < 0.001), PPE (*p* = 0.022), EAT (*p* < 0.001), and BAT (*p* < 0.001); at 72 h with PP (*p* < 0.001), EP (*p* < 0.001), BP (*p* < 0.001), EAT (*p* < 0.001), and BAT (*p* < 0.001) (Fig. 8B).

### Biological properties 2 and 3: quantification of type I collagen and osteopontin

Quantification of type I collagen secreted by osteoblastic cells cultured on the collagen membranes at 3, 5 and 7 days of incubation (expressed in percentage respect to control) in order from highest to lowest was AUBM > PPE > EP > PP > BP > BAT > EAT; observing statistically significant differences at 3 days of incubation of the collagen membrane of AUBM with PP (*p* = 0.009), EP (*p* = 0.019), BP (*p* < 0.001), EAT (*p* < 0.001), and BAT (*p* < 0.001); at 5 days with PP (*p* = 0.029), EP (0.031), BP (*p* = 0.017), PPE (*p* = 0.042), EAT (*p* < 0.001), and BAT (*p* < 0.001); and at 7 days with PP (*p* = 0.018), EP (0.029), BP (*p* = 0.012), PPE (*p* = 0.036), EAT (*p* < 0.001), and BAT (*p* < 0.001) (Table 4).

**Table 4:** Quantification of type 1 collagen secreted by osteoblastic cells plated on the membrane collagen membranes at 3, 5 and 7 days. Data is presented as % over control (Tukey test)

Collagen membranes	Day 3 (%) mean ± SD	Day 5 (%) mean ± SD	Day 7 (%) mean ± SD
PP	101.44 ± 0.78^e, f,g^	108.36 ± 0.91^e, f^	115.39 ± 1.01^e, f^
EP	101.85 ± 1.04^e, f,g^	109.57 ± 1.22^e, f^	125.44 ± 0.84^e, f^
BP	98.67 ± 1.05^d, e,f, g^	101.51 ± 1.14^e, f^	108.25 ± 1.11^e, f^
PPE	103.71 ± 2.24^c, e,f^	115.61 ± 1.04^e, f,g^	132.41 ± 1.2^e, f,g^
EAT	49.34 ± 0.49^a,b,c,d,f,g^	72.37 ± 1.12^a,b,c,d,g^	86.41 ± 0.89^a,b,c,d,g^
BAT	58.47 ± 0.75^a,b,c,d,e,g^	82.53 ± 1.11^a,b,c,d,g^	99.33 ± 0.99^a,b,c,d,g^
AUBM	105.76 ± 1.29^a,b,c,e,f^	123.61 ± 0.86^a,b,c,d,e,f^	156.34 ± 0.99^a,b,c,d,e,f^

^a^ Significant differences compared with PP

^b^ Significant differences compared with EP

^c^ Significant differences compared with BP

^d^ Significant differences compared with PPE

^e^ Significant differences compared with EAT

^f^ Significant differences compared with BAT

^g^ Significant differences compared with AUBM

The quantification of osteopontin secreted by osteoblastic cells plated on the collagen membranes at 3, 5 and 7 days of incubation (expressed in percentage respect to the control) in order from highest to lowest was AUBM > PPE > EP > PP > BP > BAT > EAT; observing statistically significant differences at 3 days of incubation of the collagen membrane of AUBM with BP (*p* = 0.030), EAT (*p* < 0.001), and BAT (*p* < 0.001); at 5 days with BP (*p* = 0.013), EAT (*p* < 0.001), and BAT (*p* < 0.001); at 7 days with EAT (*p* < 0.001), and BAT (*p* < 0.001) (Table 5).

**Table 5:** Quantification of osteopontin secreted by osteoblastic cells plated on the membrane collagen membranes at 3, 5 and 7 days. Data is presented as % over control (Tukey test)

Collagen membranes	Day 3 (%) mean ± SD	Day 5 (%) mean ± SD	Day 7 (%) mean ± SD
PP	141.31 ± 4.56^e, f^	174.72 ± 3.36^e, f^	185.91 ± 5.07^e, f^
EP	143.73 ± 7.01^e, f^	176.75 ± 5.46^e, f^	189.87 ± 5.09^e, f^
BP	135.51 ± 4.24^e, f,g^	160.44 ± 6.78^d, e,f, g^	171.25 ± 12.41^e, f^
PPE	147.72 ± 9.33^e, f^	179.17 ± 6.29^c, e,f^	191.59 ± 5.08^e, f^
EAT	62.94 ± 2.16^a, b,c, d,g^	95.06 ± 3.69^a, b,c, d,g^	111.87 ± 7.03^a, b,c, d,g^
BAT	73.35 ± 2.69^a, b,c, d,g^	101.67 ± 6.67^a, b,c, d,g^	120.46 ± 15.05^a, b,c, d,g^
AUBM	154.46 ± 9.39^c, e,f^	181.71 ± 9.41^c, e,f^	195.93 ± 4.98^e, f^

^a^ Significant differences compared with PP

^b^ Significant differences compared with EP

^c^ Significant differences compared with BP

^d^ Significant differences compared with PPE

^e^ Significant differences compared with EAT

^f^ Significant differences compared with BAT

^g^ Significant differences compared with AUBM

### Biological properties 4: ALP activity

**Fig. 9:**
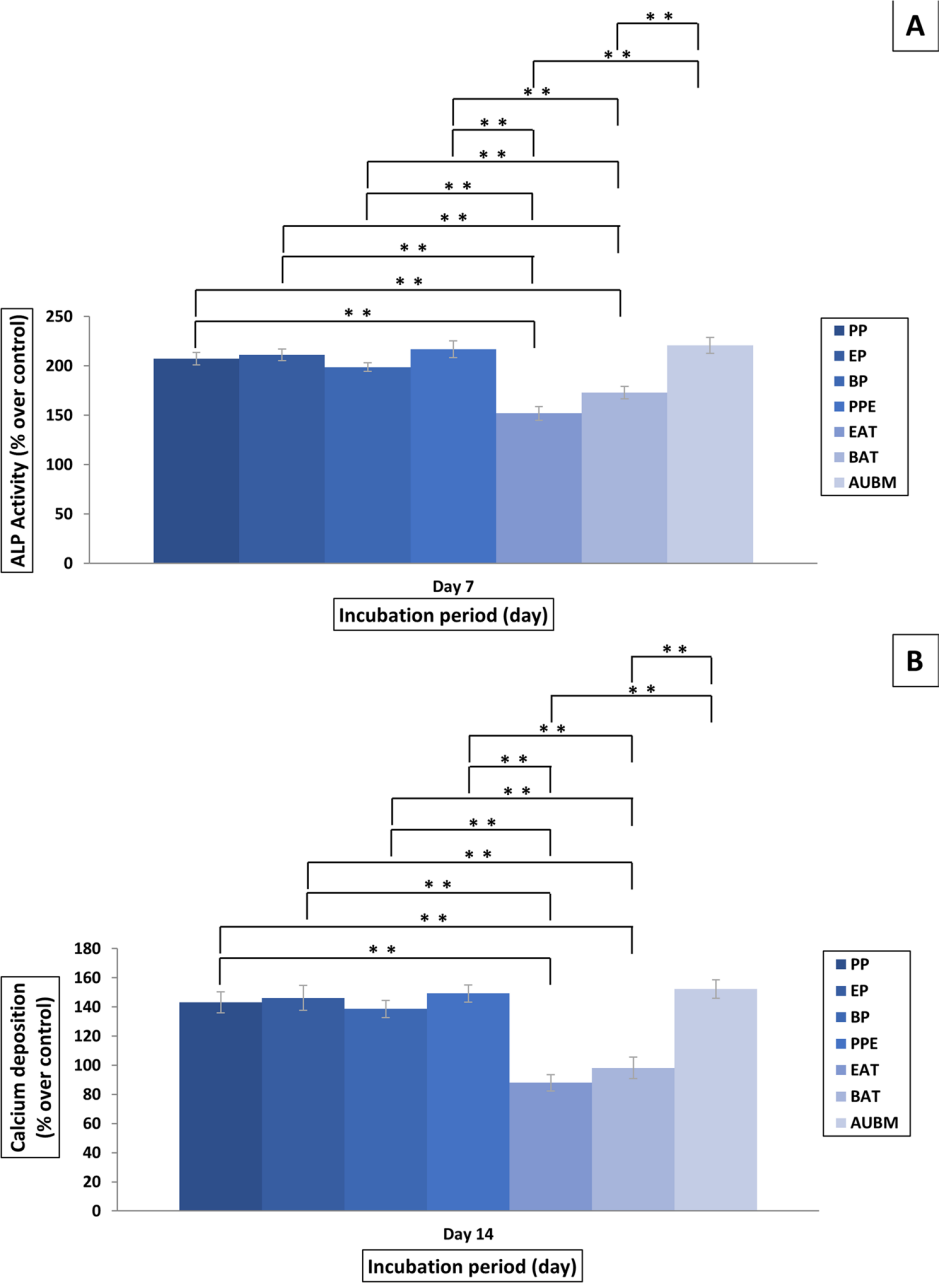
**A**: ALP activity (* *p*<0.05; ** *p*<0.001). **B**: Calcium deposition (* *p*<0.05; ** *p*<0.001)

At 7 days of culture, the enzymatic ALP activity (expressed as a percentage respect to control) in order from highest to lowest was AUBM > PPE > EP > PP > BP > BAT > EAT, although it was very similar in AUBM, PPE, EP, PP, and BP; in fact statistically significant differences (*p* < 0.001) were observed for these five membranes when compared to BAT or EAT (Fig. 9A).

### Biological properties 5: alizarin red staining (calcium deposition)

At 14 days of culture, the calcium deposition (expressed in percentage respect to control) in order from highest to lowest was AUBM > PPE > EP > PP > BP > BAT > EAT, although it was very similar in AUBM, PPE, EP, PP, and BP; in fact statistically significant differences (*p* < 0.001) were observed for these five membranes when compared to BAT or EAT (Fig. 9B).

## Discussion

The present study compared the physical (surface morphology, hydrophilic property, degradation ratio and thermal stability), mechanical (tensible strength and elongation), and biological (cell viability, quantification of type I collagen, quantification of osteopontin, ALP activity and calcium deposition) properties of a collagen membrane of AUBM with six collagen membranes of porcine (PP and PPE), equine (EP and EAT) and bovine (BP and BAT) origin.

Regarding surface morphology, the collagen membrane of AUBM showed on both surfaces (smooth and rough) regular and closely arranged collagen bundles, as did the BP, EP, EAT and BAT membranes, while the PPE membranes showed on both surfaces slightly irregular and loosely arranged collagen bundles. In our FESEM analysis, only the BP membranes showed collagen bundles that showed collagen bundles on both surfaces that were interwoven to form 3D grid with uniform pores. Our results are in agreement with those obtained by Shi et al., in 2023 [34] who analysed with scanning electron microscopy (SEM) the surface morphology of the collagen membrane of PPE, bovine dermis, and BP. The results obtained indicated that also the bovine dermis membrane showed regular and arranged collagen bundles like our AUBM, EP, EAT and BAT membranes; that the PPE membranes showed also in both surfaces collagen bundles that were slightly irregular and loosely arranged; and that the BP membranes showed in both surfaces collagen bundles interwoven, forming a 3D grid with regular pores. However, it appears that the arrangement of the collagen arranged closely, loosely, or with pores may influence some physical properties as a hydrophilic but not the rest of the physical, mechanical or biological properties, where the most morphologically influential variable is not so much the arrangement of the collagen bundles, but their homogeneous arrangement throughout the membrane [57]. In our study, all seven membranes studied had a homogeneous collagen arrangement (closely, loosely or with pores) across the entire surface of each of the membranes analysed.

The hydrophilic properties of the collagen membranes (in both surfaces) were studied using the test of contact angles. This device makes it possible to quantify the relationship between the adhesive forces between the liquid and the membrane and the cohesive forces of the liquid; thus, when the adhesive forces with the membrane surface are greater than the cohesive forces, the contact angle is less than 90º, which indicates the hydrophilic capacity of the membranes. The first thing to note is that, of the seven collagen membranes studied, only the EAT and PP membranes showed contact angles above 90º on both the smooth and rough surfaces, indicating that they are not very hydrophilic. Secondly, we observed that in all seven membranes studied the contact angles were lower on the rough surface compared to the smooth surface, these results are related to the clinical indications for the orientation of collagen membranes in ROG, since surfaces with low contact angles may increase cell adhesion and tissue regeneration [58, 59], in this sense in ROG the smooth surface (less hydrophilic) should be in contact with the soft tissue and not the bone, while the rough surface (more hydrophilic), should be in contact with the bone. Thirdly, we observed that PPE collagen membranes were the most hydrophilic with statistically significant differences with the other six collagen membranes on both surfaces, since the contact angle of the drop of distilled H_2_O could not be measured at 4 s on any of the surfaces, because it was absorbed in less than 4 milliseconds. In this regard, Shi et al., [34] also observed that PPE membranes were much more hydrophilic (on both surfaces), when compared to collagen membranes from bovine dermis and BP, although these authors did not indicate at how many seconds the measured the contact angles. However, among pericardium materials of porcine, equine and bovine origins, the PP is likely to be the most hydrophobic materials, this is possible because its surface morphology shows regular and closely arranged collagen bundles with honeycomb features. Finally, the collagen membrane of AUBM showed in both surfaces (smooth and rough) to be more hydrophilic than PP, EP, BP, EAT and BAT. Our results coincide with those obtained by Rodrigues et al., in 2020 [60] who compared the hydrophilic properties of polyurethane with another one made of polyurethane but rich in collagen and elastin and observed that the contact angles were much smaller in the membrane rich in collagen and elastin, with a composition very similar to the collagen membrane of AUBM [61].

In relation to degradation ratio in artificial saliva studying the degradation weight loss at 7, 14 and 21 days we observed that ranked from lowest to highest was AUBM < PPE < EP < BP < BAT < PP < EAT. Taking into account that the average degradation time of resorbable collagen membranes in in vivo studies ranges from 8 to 12 weeks [14, 62], our results showed that even the membrane with most weight loss (%) at 21 days, the EAT membrane, had a weight loss of less than 15% of its initial weight, so we can expect that all of them would remain completely undegraded, at least until 12 weeks. In relation to the low degradation ratio of AUBM and PPE membranes, authors such as Camacho-Alonso et al., in 2021 used AUBM as scaffold for neonatal myoblasts to repair volumetric muscle loss in hemiglossectomized Lewis rats, their histological and immunohistochemical findings showed evidence of AUBM remnants 42 days after the treatments; similarly in relation to AUBM collagen membranes, Shi et al., [34] observed that the order of lowest to highest degradation ratio in artificial saliva at 7, 14 and 21 days was PPE, bovine dermis, and BP.

With regard to the thermal stability, the TG curves of weight loss (%) as a function of the temperature were very similar in the seven membranes studied, observing three peaks in temperature where there was a higher percentage of weight loss in all membranes at 80º C, 300º C and 550º C. When comparing the weight loss (%) in these three temperature peaks, they were very similar in all membranes, with no statistically significant differences. These three peaks of collagen degradation as a function of temperature were described by Qi et al., in 2018 [63] and correspond to the loss of physisorbed water below 100º C, collagen breakdown, around approximately 300º C, and collagen combustion around 550º C. Similar results were observed by Zhu et al., in 2023 [64] when comparing the TG curves of weight loss (%) collagen membranes from PPE and bovine dermis. These authors found very similar TG curves in both membranes, with the same three types of temperature where a higher percentage of weight loss occurred.

When we analysed tensile strength (MPa) of the dry and wet membranes, the first thing we observed was that tensile strength was lower when the membranes were immersed in artificial saliva for 2 min prior to testing. This is a fact to be taken into account by clinicians, who should be aware that in addition to the fact that resorbable collagen membranes have lower tensile strength than non-resorbable membranes, when wetted with the patient’s saliva, they reduce this tensile strength [65]. Our results indicated that the membranes with the highest tensile strength in dry were EAT membranes, and in wet were BAT membranes; the membrane with the lowest tensile strength in dry and wet was PPE. Similar results were observed by Shi et al., [34] where PPE membranes had lower tensile strength than BP and bovine dermis membranes. Similarly, Zhu et al., [64] observed that PPE membranes had less tensile strength than bovine dermis. Raz et al., [65] also observed less tensile strength in PPE membranes when compared to another two porcine collagen membranes, Remaix™ (Matricel GmbH, Herzogenrath, Germany) and Ossix Plus^®^ (Datum Dental Biotech, Lod, Israel). Finally, the collagen membrane of AUBM showed a tensile strength in dry of more than 20 MPa being superior to EP, BP, PP, PPE membranes. Similar results in tensile strength of AUBM were observed by Garribloli et al., in 2020 [66] who studied the mechanical properties of AUBM for its application in tissue-engineered bladder reconstruction.

However, within the mechanical properties studied (tensile strength and elongation), it should be noted that in collagen membranes used for ROG it is very important that they have high elongation, regardless of their tensile strength, since the MPa to which the membranes are subjected in in vitro tests are so high that they are unlikely to be reproduced in a real clinical situation [34]. Elongation (%) is a crucial index to characterise the softness, plasticity, and elasticity of collagen fibres in the membranes. It is this physical property that will allow the membranes to have sufficient elasticity to be able to cover different sized and morphologies of bone defects, and to be sutured or fixed without being subjected to high stresses or tearing [65]. Our results indicated that, when collagen membranes were hydrated, regardless of the collagen source from which they were obtained (different anatomical locations and animal origin), they all increased their capacity for elongation. Comparable results were obtained by Shi et al., [34] when hydrating collagen membranes from PPE, bovine dermis, and BP. Finally, the collagen membrane of AUBM showed that its elongation (%) went from 19.86 ± 16.32 to 225.78 ± 16.32, being the most elastic of the seven membranes studied. This is due to its richness in elastin [67], and that the AUMB extracellular matrix is composed of collagens type I, III, IV, and VI, and elastic fibres [66]. However, concerns remain regarding the potential impact of their comparatively weaker tensible strength on their practical application in actual clinical settings.

Regarding the biological properties (cell viability, quantification of type I collagen, quantification of osteopontin, ALP activity, and calcium deposition) after seeding human osteoblasts on the membranes, the order from the best to worst biological properties was the same in the 5 variables studied: AUBM > PPE > EP > PP > BP > BAT > EAT; which indicated that the AUBM membranes had an important action on osteoblastic and osteogenic potential. AUBM is known as a scaffold in tissue engineering where numerous cell lines have been cultured because it contains various molecules like cell adhesion proteins, growth factors, and enzymes [46]. AUBM contains a high concentration of growth factors, including platelet-derived growth factor BB (PDGF-BB), vascular endothelial growth factor (VEGF), keratinocyte growth factor (KGF), transforming growth factor beta 1 (TGF-beta 1), insulin-like growth factor (IGF), epidermal growth factor (EGF), and basic fibroblast growth factor (bFGF) [68]. These bioactive molecules create an optimal environment for osteoblast growth, proliferation, and migration [69]. In addition to these compositional characteristics, which made the collagen membrane of AUBM the one in which human osteoblast cell proliferation increased the most at 24, 48 and 72 h of incubation; we observed that at 3, 5, and 7 days of incubation, using the time points proposed by Maques et al., in 2023 [70] to quantify the levels of type I collagen and osteopontin secreted by cells cultured on the membranes, the highest expression of both occurred in the membranes of AUBM. Type I collagen is a protein that needs to be expressed by osteoblasts during the formation of mineralised bone matrix as well as for hydroxyapatite crystal nucleation. In addition, type I collagen needs to be synthesised and expressed for subsequent sequential expression of ALP, osteocalcin and bone sialoprotein [71]. Osteopontin is a non-collagenous protein also known as bone/sialoprotein I (BSP-1 or BNSP), is a 33-kDa glycoprotein that belongs to the small integrin-binding ligand N-linked glycoprotein (SIBLING) family. It is recognised as a key marker of osteogenic differentiation, as it modulates osteoblast functions through receptor signalling and also plays an important role in bone matrix mineralisation by helping to control the formation of hydroxyapatite crystals [72]. Finally, in relation to AUBM having a significant osteogenic potential on osteoblasts, we observed that ALP activity at 7 days, using the time point proposed by Felice et al., [37], and the calcium deposition at 14 days, using the time point proposed by Pierfelice et al., [73], were the highest when compared to the other six membranes studied.

One of the main limitations of the study was the inability to compare the results with other published research, as this study is the first to investigate the physical, mechanical, and biological properties of AUBM for its possible use in GBR. Another limitation of this study is that it is simply an in vitro study and cannot be fully translated to clinical situations. In this regard, additional in vivo studies are required to compare the effectiveness of the AUBM collagen membrane in GBR with those of PP, EP, BP, PPE, EAT, and BAT. Moreover, although saliva is commonly used as a degradation medium in biomaterials research, it does not fully replicate the complex in vivo environment, which includes contact with blood, mononuclear cells, and matrix metalloproteinases.

## Conclusions

In conclusion, the AUBM collagen membrane showed similar physical and mechanical properties to the other six collagen membranes of porcine, equine and bovine origin. However, when wetted, they were the membranes with the highest elongation capacity, and similar properties to PPE membranes in promoting osteogenesis. In this sense, AUBM could be an ideal collagen source for the manufacture of collagen membranes for GBR, but further clinical studies are needed.

## Data Availability

No datasets were generated or analysed during the current study.

## Abbreviations

Ala: Alanine
AUBM: Urinary bladder acellular matrix
BAT: Bovine Achilles tendon
BP: Bovine pericardium
EAT: Equine Achilles tendon
EP: Equine pericardium
e-PTEF: Expanded polytetrafluorethylene
GAGs: Glycosaminoglycans
GBR: Guided Bone Regeneration
Gly: Glycine
GTR: Guided Tisular Regeneration
Hyp: Hydroxyproline
PDO: Poly(ester-esther) polydioxanone
PEG: Polyethylene glycol
PLC: Polycaprolactone
PLGA: Poly(L-lactide-co-glycolide)
PLLA: Poly(L-lactide)
PP: Porcine pericardium
PPE: Porcine peritoneum
Pro: Proline
PTFE: Polytetrafluorethylene
